# The effect of platelet–rich plasma on clinical outcomes of the surgical treatment of periodontal intrabony defects: A systematic review and meta–analysis

**DOI:** 10.1186/s12903-016-0261-5

**Published:** 2016-08-17

**Authors:** Xinshan Hou, Jingwen Yuan, Absijiang Aisaiti, Yuan Liu, Jin Zhao

**Affiliations:** 1 The Oral Medicine Clinical Center, The First Affiliated Hospital of Xinjiang Medical University, No. 137 South Liyushan Road, Urumqi, 830054 People’s Republic of China; 2 Stomatology Disease Institute of Xinjiang Uyghur Autonomous Region, No. 137 South Liyushan Road, Urumqi, 830054 People’s Republic of China

**Keywords:** Periodontal disease, Platelet-rich plasma, Randomized controlled trial, Meta-analysis

## Abstract

**Background:**

Studies investigating the use of platelet–rich plasma (PRP) in the treatment of intrabony defects have yielded mixed results. The aim of our study was to evaluate the efficacy of PRP by comparing clinical attachment level (CAL) and pocket depth (PD) for patients who received PRP as an adjunct to periodontal intrabony defect therapy with those for patients who did not. We also analyzed the influence of guided tissue regeneration (GTR) and different study designs (parallel and split–mouth studies) on the clinical outcomes of intrabony defects.

**Methods:**

We performed a systematic review of articles published in any language up to June 7, 2015 by searching PubMed, Embase, Web of Science, and the Cochrane Central Register of Controlled Trials. We included only randomized controlled clinical trials (RCTs) that compared clinical outcomes between patients who received PRP as an adjunct to periodontal intrabony defect therapy and patients who did not. We combined data from randomized trials to assess clinical outcomes using a random–effects model.

**Results:**

Of the 307 abstracts that were initially identified, 12 RCTs related to the treatment of periodontal intrabony defects were included in the final analysis. Clinically and significantly greater CAL gains and PD reductions were observed in subjects who received PRP as an adjunct to periodontal intrabony defect therapy than in subjects who did not (CAL: WMD 0.76 mm, 95 % CI = 0.34 to 1.18 mm, *P =* 0.0004; PD: WMD 0.53 mm, 95 % CI = 0.21 to 0.85 mm, *P =* 0.001). Subgroup meta-analyses of patients who underwent GTR demonstrated that this approach did not significantly affect treatment outcomes (CAL: WMD 0.08 mm, 95 % CI = −0.30 to 0.46 mm, *P =* 0.67), as indicated by a comparison with patients who did not undergo GTR (CAL: WMD 1.22 mm, 95 % CI = 0.88 to 1.57 mm, *P <* 0.00001). Univariate meta-regression analyses revealed that the use of GTR explained the heterogeneity among the included studies (*P <* 0.05).

**Conclusions:**

Within its limitations, this review suggests that PRP may be beneficial as an adjunct to graft materials for the treatment of periodontal intrabony defects, except in cases involving the use of GTR.

## Background

Periodontitis is a disease of the periodontium characterized by an irreversible loss of attachment to the connective tissue and supporting alveolar bone [1]. Periodontitis will continue to progress if no intervention is undertaken and will ultimately result in early tooth loss. Current therapeutic modalities to restore the disrupted periodontium, such as conventional open flap debridement (OFD), have shown limited potential to achieve the desired results [2].

The key to tissue regeneration is to stimulate a cascade of healing events that, if coordinated, can result in the completion of integrated tissue formation. Such modulators include the use of growth factors, the application of extracellular matrix proteins and attachment factors, and the use of bone morphogenetic proteins [3]. The potential role of polypeptide growth factors (PGFs) in periodontal regeneration is currently a focus of research. Among the PGFs, platelet-derived growth factor (PDGF) and transforming growth factor-β (TGF-β) have been the most extensively studied in terms of periodontal regeneration. These components are known to facilitate bone regeneration after bone grafting by enhancing neoangiogenesis, cellular chemotaxis and mitosis, promoting stem cell proliferation, and increasing osteoconduction via the fibrin network [4]. For decades, there has been a growing interest in the use of platelet-rich plasma (PRP) for the treatment of periodontal intrabony defects. PRP is a concentrated source of autologous platelets that is enriched with several growth factors, including PDGF, transforming growth factor-1 (TGF-1), transforming growth factor-2 (TGF-2), vascular endothelial growth factor (VEGF), insulin-like growth factor-1 (IGF-1), insulin-like growth factor-2 (IGF-2), fibroblast growth factor-β (FGF-β) and epithelial growth factor (EGF). All of these hormones are secreted by platelets to initiate wound healing [5]. Some studies [6–11] have suggested that following coagulation, the PRP preparation exhibits a “sticky consistency” that may improve the clinical handling properties of the combination of PRP and the graft material, thereby enhancing wound stability.

Recently published systematic reviews and meta-analyses [12, 13] on this topic have demonstrated the beneficial effect of PRP in the treatment of intrabony defects. However, high heterogeneity among the examined studies rendered it difficult to draw clear interpretations. We will explore the sources of heterogeneity between studies through subgroup meta-analyses and a meta-regression. The aim of our study was to evaluate the efficacy of PRP in the surgical treatment of periodontal intrabony defects by comparing clinical outcomes between patients who received PRP as an adjunct to periodontal intrabony defect therapy and those who did not.

## Methods

### Search strategy

This systematic review was performed in accordance with the guidelines of the Preferred Reporting Items for Systematic reviews and Meta-Analyses (PRISMA) statement and the Cochrane Handbook [14, 15]. Four electronic databases (PubMed, Embase, Web of Science and the Cochrane Central Register of Clinical Trials) were searched using the following keywords: (“platelet rich plasma” OR “PRP” OR “autologous platelet concentrate” OR “platelet gel”) AND (“periodontal atrophy” OR “periodontal defects” OR “intrabony defects” OR “infrabony defect” OR “periodontal osseous defects”). The search was limited to clinical trials involving human subjects with no restrictions with respect to language. All databases were searched from their inception to June 2015. The bibliographies of all original research and review articles identified to be relevant to the subject were scanned for possible additional studies. The literature search was performed by two examiners (X.H. and J.Y.).

### Study selection criteria

Studies were selected if they fulfilled the following inclusion criteria: 1) a randomized controlled clinical trial (RCT) in which an intervention group receiving PRP was compared with a control group not receiving PRP; 2) the patients included in the RCT had no systemic illness or abnormal platelet counts that could affect the clinical outcome of periodontal therapy; and 3) a follow-up period of at least 6 months.

The exclusion criteria included the following: 1) an inadequate comparison of the results of PRP for the treatment of periodontal intrabony defects; 2) PRP administered to both the intervention and control groups; 3) the use of a biologic material that would hamper meaningful comparisons; or 4) other article types, such as reviews, case reports, and animal studies.

### Data extraction and quality assessment

The characteristics of the included studies were extracted by two reviewers (X.H. and J.Y.), and the relevant data from the studies that met the inclusion criteria were extracted independently. Any discrepancy was resolved by discussion. The following characteristics of each included study were recorded: characteristics of the trial (first author’s last name, publication year, study design, number of patients, number of defect sites, length of follow-up, and evaluation indicators); intervention (types of bone substitutes and parameters of PRP preparation and application); and outcome measures.

The risk of bias was evaluated independently by two reviewers (A.A. and Y.L.), and any disagreements were resolved by a third reviewer (X.H.). The quality of the selected RCTs was assessed using the Risk of Bias tool according to the Cochrane Handbook for Systematic Reviews of Interventions (Version 5.1.0) [15]. The selected RCTs were assessed using the following criteria: sequence generation, allocation concealment, masking of the examiner, incomplete outcome data, free of selective outcome reporting, and other sources of bias.

### Outcome variables and statistical analyses

For studies evaluating the effect of PRP in the treatment of intrabony defects, the change in CAL from the initial diagnosis to the final follow-up was our primary outcome variable. The change in probing depth between baseline and the final follow-up was considered the secondary outcome variable.

The meta-analysis was performed on similar studies that only evaluated the difference between the intervention and control groups regarding the adjunctive use of PRP. First, the pooled weighted mean difference of the outcome variables was estimated using Review Manager Version 5.3 (The Nordic Cochrane Centre, The Cochrane Collaboration, Copenhagen, Denmark). The results are expressed as the mean differences for continuous outcomes using the random-effects model. Forest plots were constructed to graphically represent the difference in outcomes between the intervention and control groups. The significance level for this meta-analysis model was 0 · 05. The statistical heterogeneity among the included studies was evaluated using the chi-square (*χ*^2^) and I^2^ tests. Publication bias was evaluated through funnel plots and Egger’s test using STATA software (STATA/SE 12; Stata Corp, College Station, TX, USA). For this test, a *P* value of less than 0.1 shows significant asymmetry and therefore publication bias [16]. Second, we performed a subgroup meta-analysis of CAL to determine the effects of the use of GTR and of different study designs. We performed subgroup analyses for these two specific moderators due to their well-known clinical implications and statistical effects [17, 18]. On the one hand, a prior meta-analysis [17] demonstrated that the proven efficacy of GTR in regenerative periodontal procedures could mask the effects of a platelet concentrate. On the other hand, a study [18] published in 2009 suggested that it is advisable to meta-analyze split-mouth and parallel-group trials separately as subgroups to investigate their systematic differences.

Third, we explored other sources of heterogeneity in studies through a meta-regression analysis [19, 20]. We performed a separate univariate meta-regression analysis using STATA software; multivariate meta-regression analysis was not performed due to the inclusion of an inadequate number of studies. A random-effects model with a restricted maximum-likelihood estimator was used to synthesize effect size across studies. The variables we selected included not only the use of GTR and study design but also the type of control (allograft, xenograft, or artificial bone), which was reported in all of the included studies and may be a potential source of heterogeneity.

## Results

**Fig. 1:**
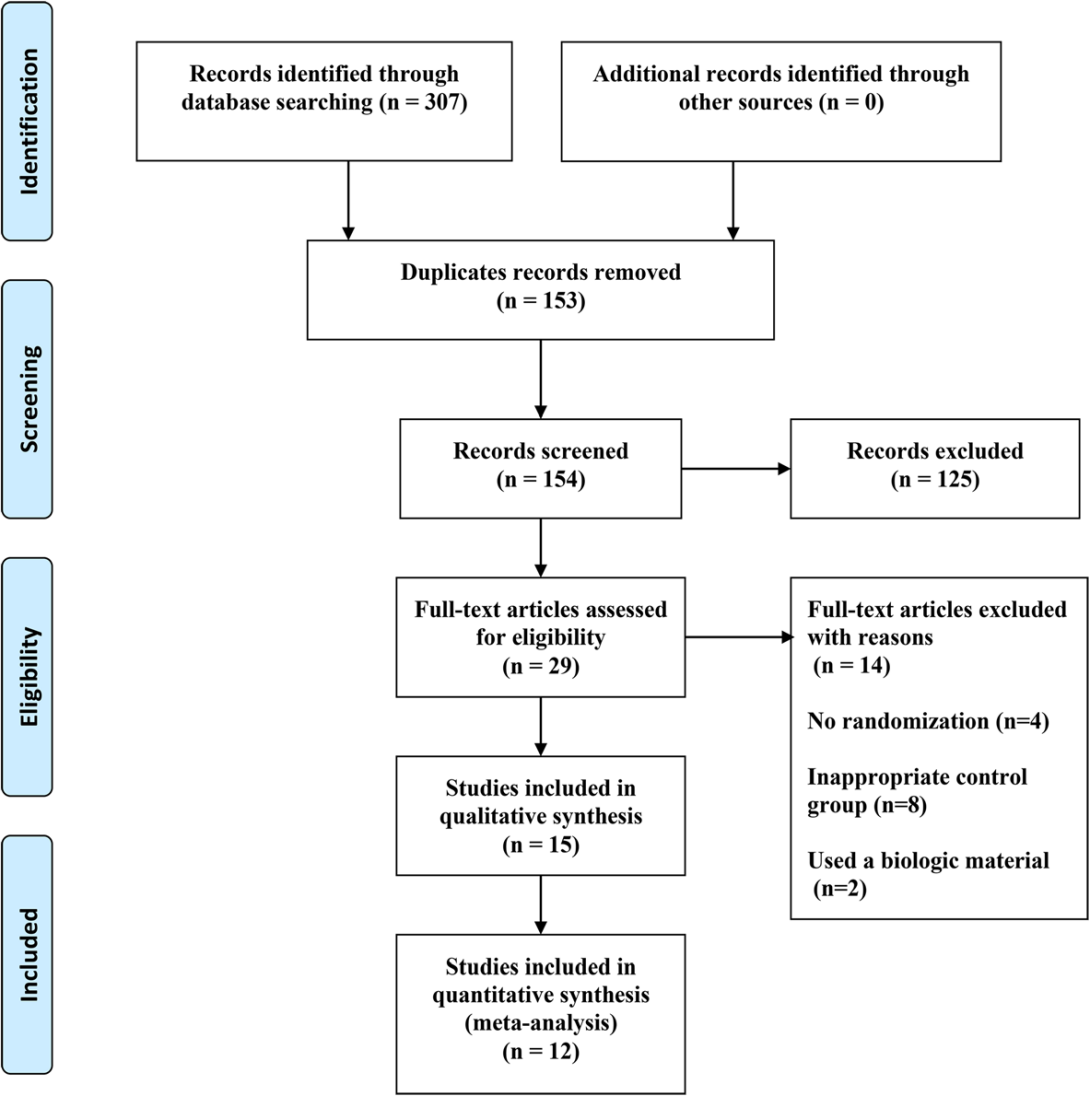
Flow chart of the study selection process

A total of 307 studies were identified after searching four databases. After screening the titles and abstracts, 125 studies were extracted. The full texts of 29 studies were reviewed, and 14 additional studies were excluded. Fifteen studies were included in this systematic review, and twelve of these were included in the final analysis. The study selection process is shown in Fig. 1. A summary of the excluded studies [9, 21–33] and the reasons for the exclusion of each study are listed in Table 1. The main characteristics of the included studies are summarized in Table 2.

**Table 1:** Summary of the excluded studies and the reason for their exclusion

Reference	Reason for exclusion
Camargo et al. 2005 [9]	Inappropriate control group (use of OFD instead of GTR + BM)
Rodrigues et al. 2011 [21]	Inappropriate control group (use of PRP instead of ABM)
Yilmaz et al. 2011 [22]	Inappropriate control group (use of PPP + BDX instead of BDX)
Yilmaz et al. 2010 [23]	Inappropriate control group (use of PRP + BDX instead of BDX)
Pradeep et al. 2009 [24]	Inappropriate control group (use of PRP instead of ABM/P-15)
Yamamiya et al. 2008 [25]	Inappropriate control group (use of HA + PRP instead of HA + HCP)
Ilgenli et al. 2007 [26]	Inappropriate control group (use of PRP instead of DFDBA)
Camargo et al. 2002 [27]	Inappropriate control group (use of GTR instead of GTR + BM)
Pradeep et al. 2012 [28]	Inappropriate control group (use of OFD instead of bone graft)
Yilmaz et al. 2009 [29]	Not a randomized controlled clinical trial
Camargo et al. 2009 [30]	Not a randomized controlled clinical trial
Czuryszkiewicz-Cyrana et al. 2006 [31]	Not a randomized controlled clinical trial
Döri et al. 2008 [32]	Used a biologic material (EMD)
Döri et al. 2013 [33]	Used a biologic material (EMD)

*ABM* anorganic bone mineral, *BDX* bovine-derived xenograft, *P*-15 peptide-15, *HCP* human cultured periosteum, *OFD* open flap debridement, *HA* hydroxyapatite, *DFDBA* demineralized freeze-dried bone allograft; *BM* bovine-derived porous bone mineral, *EMD* enamel matrix derivative

**Table 2:** Characteristics of the randomized trials reporting on the treatment of periodontal intrabony defects

Authors and publication year	Design	Country	Patients	Sites	Treatment		Site		Defects (walls)	Follow-up	Effect	Evaluation
					Intervention	Control	Intervention	Control				
Gupta G et al. 2014 [34]	Split-mouth	India	10	20	PRP + HA	HA	10	10	1, 2, 3	12 mo	Positive	BOP, PD, CAL,
Okuda K et al. 2005 [11]	Parallel	Japan	70	70	PRP + HA	HA	35	35	2, 3	12 mo	Positive	GI, BOP, PD, CAL, GR, DF
Hanna R et al. 2004 [35]	Split-mouth	US	13	26	PRP + BDX	BDX	13	13	2, 3	6 mo	Positive	PD, CAL, GI, PI, REC, BOP
Ouyang XY et al. 2006 [36]	Split-mouth	China	10	17	PRP + ABB	ABB	9	8	2, 3	12 mo	Positive	PI, PD, CAL, REC, Bone defect fill
Döri et al. 2009 [37]	Parallel	Hungary	30	30	PRP + ABB	ABB	15	15	1,2	13 mo	None	PD, GR, CAL, PI, GI, BOP,
Demir et al. 2007 [38]	Parallel	Turkey	29	29	PRP + BG	BG	15	14	1, 2, 3	9 mo	None	PD, CAL, GR, PI, GI, BOP,
Agarwal et al. 2014 [39]	Split-mouth	India	24	48	PRP + DFDBA	DFDBA	24	24	2, 3	12 mo	Positive	PD, CAL, REC
Piemontese et al. 2008 [40]	Parallel	India	60	60	PRP + DFDBA	DFDBA	30	30	2, 3	12 mo	Positive	GI, PI, PD, CAL, BOP, REC,
Harnack et al. 2009 [41]	Split-mouth	Germany	22	44	PRP + β-TCP	β-TCP	22	22	2	6 mo	Positive	GI, PI, PD, CAL, BOP,
Özdemir et al. 2012 [ 43]	Parallel	Turkey	14	28	PRP + β-TCP	β-TCP	14	14	2,3	7 mo	None	PD, CAL, GI, BOP,
Kaushick et al. 2007 [43]^\^	Split-mouth	India	10	20	PRP + β-TCP + HA	β-TCP + HA	10	10	2, 2–3, 3	6 mo	Positive	PD, CAL, GI, PI
Christgau et al. 2006 [44]	Split-mouth	Germany	25	50	PRP + β-TCP + GTR	β-TCP + GTR	25	25	2, 2–3, 3	12 mo	None	PI, GI, BOP, PD, GR, CAL,
Döri, Huszar et al. 2007 [45]	Parallel	Hungary	24	24	PRP + ABB + GTR	ABB + GTR	12	12	1-2, 2	12 mo	None	BOP, PD, CAL,
Döri et al. 2007 [46]	Parallel	Hungary	30	30	PRP + NBM + GTR	NBM + GTR	15	15	1-2, 2, 3	12 mo	None	PD, GR, CAL, PI, GI, BOP,
Döri et al. 2008 [47]	Parallel	Hungary	28	28	PRP + β-TCP + GTR	β-TCP + GTR	14	14	1-2, 2, 3	12 mo	Positive	PD, CAL, PI, GI, BOP, GR

*Intervention* PRP group, *HA* hydroxyapatite, *BDX* bovine-derived xenograft, *ABB* anorganic bovine bone, *BG* bioactive glass, *DFDBA* demineralized freeze-dried bone allograft, *β-TCP* β-tricalcium phosphate, *NBM* natural bone mineral

### Features of the included studies

#### Characteristics of the participants

Fifteen RCTs reported on the treatment of periodontal defects, and these studies included 524 periodontal intrabony defects treated in 399 patients (263 defects in the intervention group, 261 defects in the control group). The number of patients in each study ranged from 10 [34, 36, 43] to 70 [11] and the number of periodontal defects in each study ranged from 17 [36] to 70 [11]. Most of the studies employed common exclusion criteria, including patients with any systemic illness known to affect periodontal healing; patients exhibiting platelet deficiency, which can compromise the PRP preparation; pregnant/lactating mothers; immuno-compromised individuals; patients using drugs that may impede wound healing; patients exhibiting hypersensitivity to any medication used in the study; and individuals with poor oral hygiene. The follow-up period in these studies ranged from 6 to 12 months.

#### Characteristics of the periodontal defects

The following clinical situation reported at baseline was used in the selected studies: 1) a good level of oral hygiene (plaque index [PI] < 1); 2) the presence of an intrabony defect at a PD > 5-6 mm after phase 1 therapy (scaling and root planing [SPR]) and an intrabony component of 2–4 mm as detected on radiography; 3) no intrabony defects extending into a furcation area; and 4) no teeth displaying furcation involvement. The types of periodontal intrabony defects in the selected studies are shown in Table 2.

#### Characteristics of the interventions

Various bone substitutes (deproteinized bovine bone [35–37, 45, 46], β-tricalcium phosphate [42–44, 47], demineralized freeze-dried bone allograft [39, 40], bioactive glass [38], and hydroxyapatite [34, 11]) were combined with PRP in the selected studies. Four studies [44–47] performed supplementary guided tissue regeneration (GTR) using membranes such as expanded polytetrafluoroethylene membrane (e-PTFE) [45, 47] and bio-absorbable collagen membrane (COL) [44, 46]. The details regarding the method of PRP preparation, including the type of cell separation device, the centrifugation steps, the baseline and treatment platelet counts, and the activators of coagulation, in the selected studies are shown in Table 3.

**Table 3:** Method of platelet-rich plasma preparation in all selected randomized controlled clinical trials

Authors and publication year	Treatment		PRP preparation	Centrifugation steps	Activator (s) of coagulation	Platelet count
	Intervention	Control				
Gupta G et al. 2014 [34]	PRP + HA	HA	Not reported	Two (1200 r.p.m., 20 min & 2000 r.p.m., 15 min)	10 % CaCl_2_ mixed with human thrombin	Not recorded
Okuda K et al. 2005 [11]	PRP + HA	HA	Heraeus Labofuge 300	Two (2400 r.p.m., 10 min & 3600 r.p.m., 16 min)	0.1 g of sodium alginate	Not recorded directly; reference was made to a previous study [48], Baseline: 257 × 10^3^/μL ± 46 × 10^3^/μL
Hanna R et al. 2004 [35]	PRP + BDX	BDX	SmartPReP	Two (2400 r.p.m., 10 min & 3600 r.p.m., 15 min)	1 mL of 10 % CaCl2, mixed with 1000 United States, Units of topical thrombin	Not recorded
Ouyang XY et al. 2006 [36]	PRP + ABB	ABB	Universal 16R centrifuge	Two (1220 r.p.m., 15 min & 3600 r.p.m., 15 min)	Sterile saline solution containing 10 % CaCl_2_ mixed with 100 U/mL sterile bovine thrombin	Baseline: 189 × 10^3^/μL ± 37 × 10^3^/μL., Post treatment: 680× 10^3^/μL ± 103 × 10^3^/μL
Döri et al. 2009 [37]	PRP + ABB	ABB	Curasan PRP kit	Two (1220 r.p.m., 15 min & 3600 r.p.m., 15 min)	Sterile saline solution containing 10 % CaCl_2_ mixed with 100 U/mL sterile bovine thrombin	Not recorded directly; reference was made to a previous study [49], Post treatment: 2519.6 × 10^3^/μL ± 834.3 × 10^3^/μL
Demir et al. 2007 [38]	PRP + BG	BG	Heraeus Christ Medifuge	Two (3000 r.p.m., 10 min & 3600 r.p.m., 10 min; or 200 g, 10 min	0.3 mL of 0.025 M CaCl_2,_ mixed with blood harvested from the surgical site	Baseline: 189× 10^3^/μL ± 37 × 10^3^/μL, Post treatment: 680 × 10^3^/μL ± 103× 10^3^/μL
Agarwal et al. 2014 [39], Piemontese et al. 2008 [40]	PRP + DFDBA	DFDBA	SmartPReP	Two (2400 r.p.m., 10 min & 3600 r.p.m., 15 min)	1 mL of 10 % CaCl_2_ mixed, with 1000 United States, Units of topical thrombin	Not recorded
Harnack et al. 2009 [41]	PRP + β-TCP	β-TCP	Curasan PRP kit	Two (3169 r.p.m., 10 min & 4725 r.p.m., 15 min; or 900 g, 10 min & 2000 g, 15 min)	Blood harvested from the, surgical site	Not recorded
Özdemir et al. 2012 [42]	PRP + β-TCP	β-TCP	Curasan PRP kit	Two (2400 r.p.m., 10 min & 3600 r.p.m., 15 min)	Not recorded	Baseline: 290 × 10^3^/μL ± 86 × 10^3^/μL. Post treatment: 1075× 10^3^/μL ± 636 × 10^3^/mL
Kaushick et al. 2007 [43]	PRP + β-TCP + HA	β-TCP + HA	Not reported	Two (5000 r.p.m., 10 min & 2000 r.p.m., 10 min)	10 % CaCl_2_ mixed with an equal volume of saline.	Baseline: 200 × 10^3^/μL Post treatment: 1250 × 10^3^/μL.
Christgau et al. 2006 [44]	PRP + β-TCP + GTR	β-TCP + GTR	Spectra cell separator	Not recorded	0.5 ml of a sterile 10 % CaCl_2_ solution	Baseline: 273× 10^3^/μL ± 56 × 10^3^/μL, Post treatment: 2134 × 10^3^/μL ± 782× 10^3^/μL
Döri et al. 2007 [45], Döri et al. 2007 [46] Döri et al. 2008 [47]	PRP + ABB + GTR PRP + NBM + GTR PRP + β-TCP + GTR	ABB + GTR NBM + GTR β-TCP + GTR	Curasan PRP kit	Two (1220 r.p.m., 15 min & 3600 r.p.m., 15 min)	Sterile saline solution containing 10 % CaCl_2_ mixed with 100 U/mL sterile bovine thrombin	Not recorded directly; reference was made to a previous study [49], Post treatment: 2519.6 × 10^3^/μL ± 834.3 × 10^3^/μL

SmartPReP; Harvest Technologies Corp, Plymouth, MA, USA. Heraeu + A1:G16s Labofuge 300; Kendro Laboratory Products, Osterrode, Germany. Universal 16R centrifuge Hettich, Germany. Heraeus Christ Medifuge; Heraeus, Stuttgart, Germany. Curasan PRP kit; Curasan AG, Kleinostheim, Germany. Spectra cell separator; Cobe BCT, Lakewood, CO, USA

### Change in CAL

**Fig. 2:**
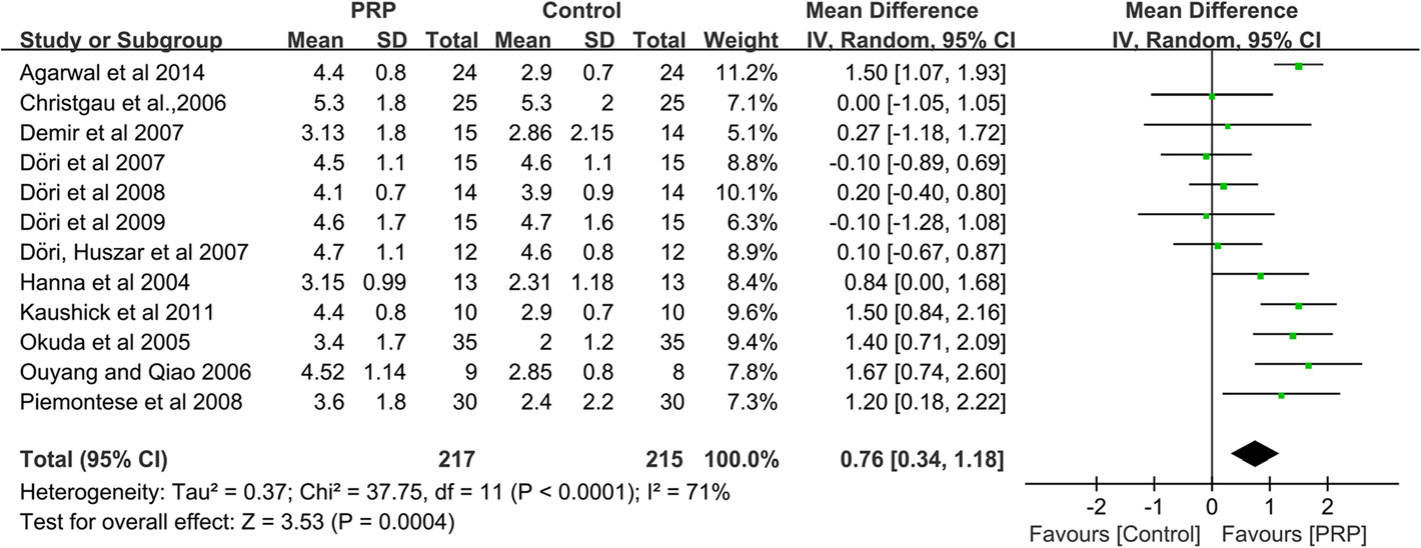
Meta-analysis of the mean difference (MD) in CAL gain due to the treatment of periodontal intrabony defects

**Fig. 3:**
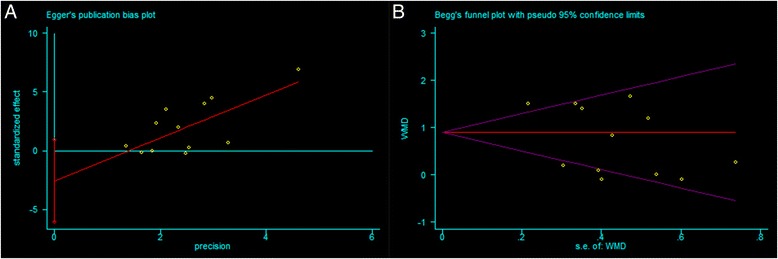
Egger’s publication bias plot **a** and Begg’s funnel plot **b** of studies that evaluated CAL gain due to the treatment of periodontal intrabony defects

The increase in CAL was significantly greater in the intervention group treated with PRP than in the control group, as determined through the random-effects model, which included twelve studies [11, 35–40, 43–47]. PRP showed a significantly positive effect on periodontal intrabony defect treatment (CAL: WMD 0.76 mm, 95%CI = 0.34 to 1.18 mm, *P =* 0.0004) (Fig. 2). No definitive publication bias was detected in the meta-analysis of the studies reporting on the change in CAL (Egger’s test *t* value = −1.64, 95%CI = −6.09 to 0.92, *P =* 0.13), and the funnel plot appeared to be symmetric, indicating an absence of publication bias (Fig. 3).

### Change in PD

**Fig. 4:**
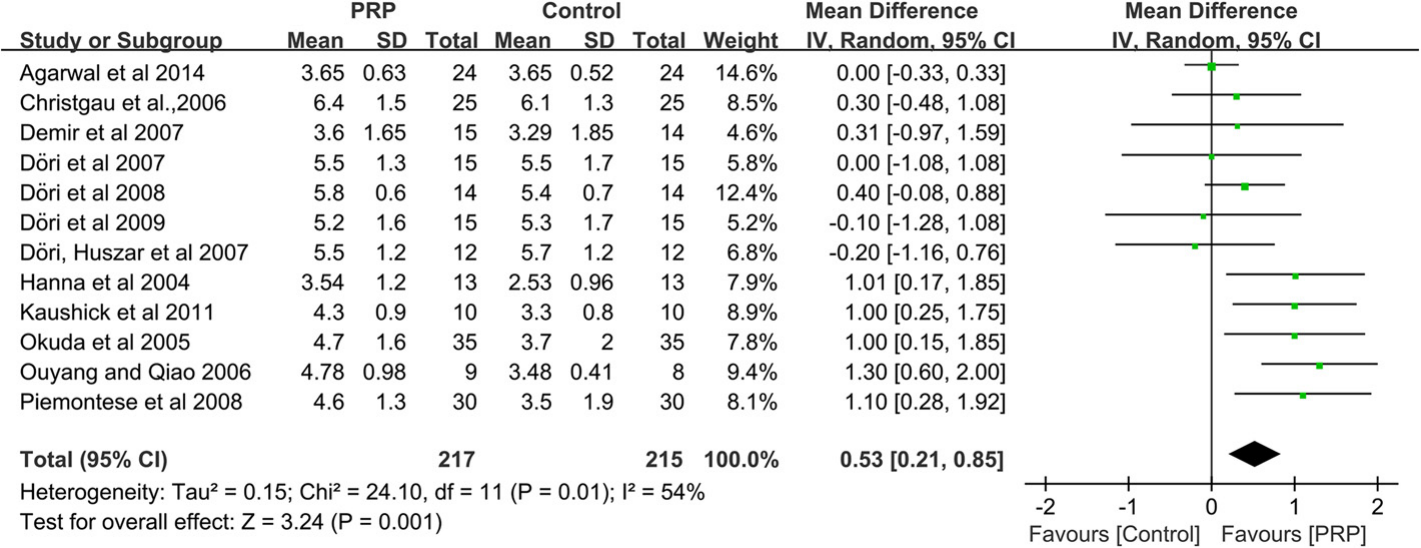
Meta-analysis of the mean difference (MD) in PD reduction due to the treatment of periodontal intrabony defects

The PD reduction was significantly greater in the intervention group treated with PRP than in the control group based on the random-effects model, which included twelve studies (PD: WMD 0.53 mm, 95%CI = 0.21 to 0.85 mm, *P =* 0.001) (Fig. 4).

### Subgroup analyses

**Fig. 5:**
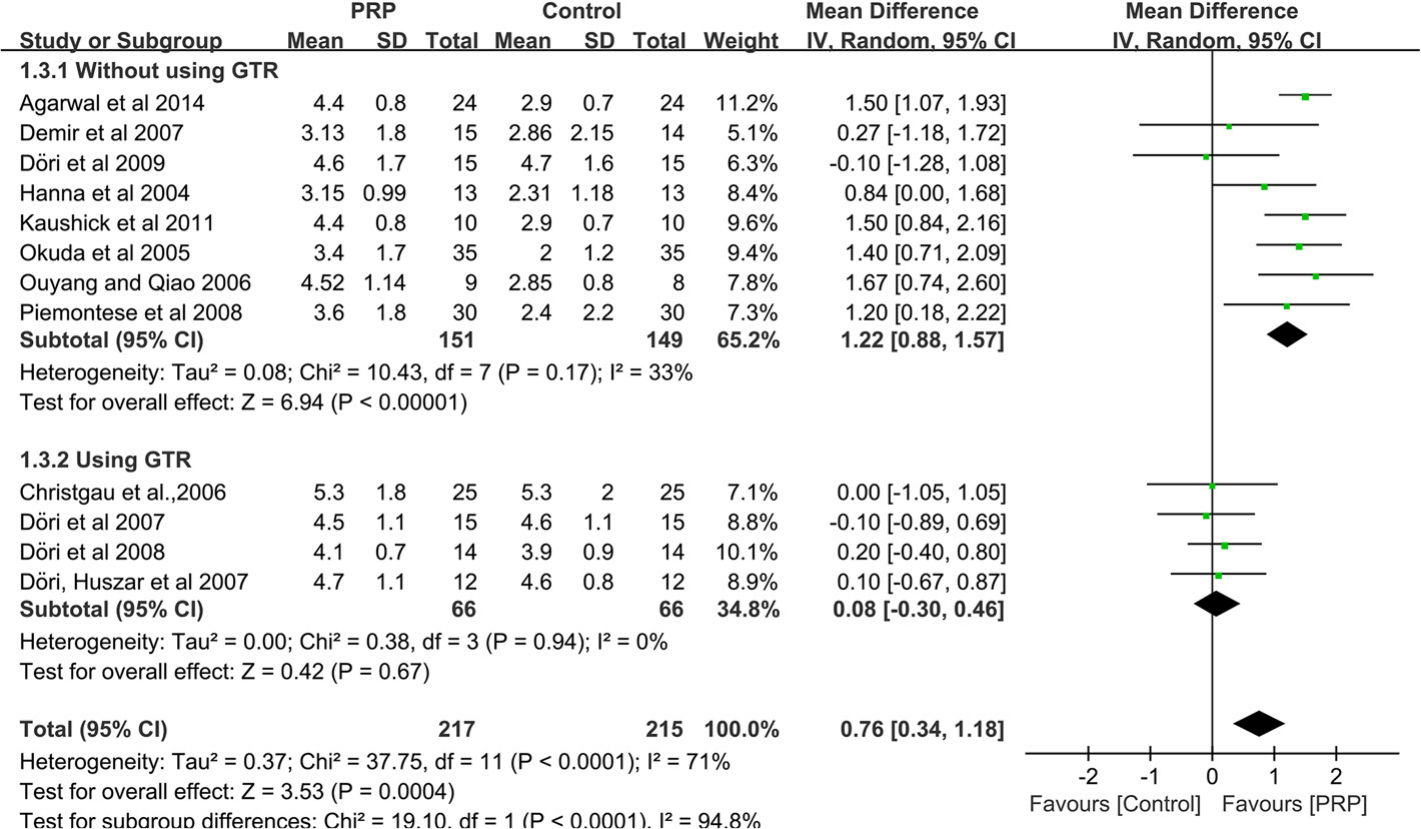
Subgroup analysis of the mean difference (MD) in CAL gain among studies that evaluated the use of PRP with or without the concomitant use of GTR

The results of our subgroup meta-analysis of the GTR technique indicated that the CAL gains of patients who underwent GTR and patients who did not undergo GTR remained significantly different. As demonstrated by the four studies [44–47] that used PRP together with GTR, PRP had an insignificant effect on the treatment (CAL: WMD 0.08 mm, 95%CI = −0.30 to 0.46 mm, *P =* 0.67). However, the eight studies [11, 35–40, 43] that used PRP without GTR showed that PRP had a significant positive effect on periodontal intrabony defect treatment (CAL: WMD 1.22 mm, 95%CI = 0.88 to 1.57 mm, *P <* 0.00001) (Fig. 5).

**Fig. 6:**
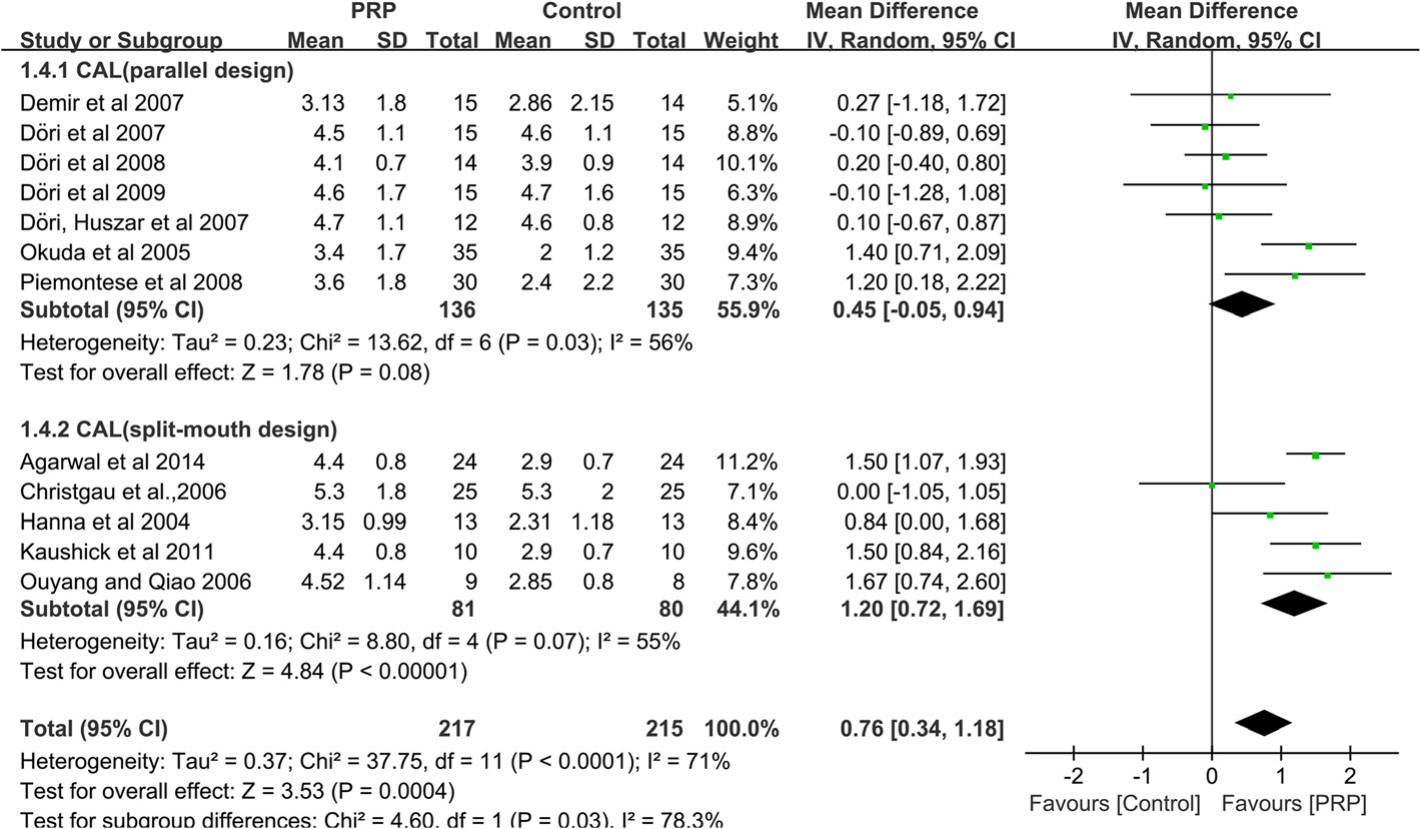
Subgroup analysis of the mean difference (MD) in CAL gain among studies that utilized a split-mouth design or a parallel design for analysis of the treatment of periodontal intrabony defects

An additional subgroup meta-analysis was performed on the study design. A significant difference in outcome was found between the different study designs. In the seven parallel-group studies [11, 37, 38, 40, 45–47], the mean difference in CAL gain between the intervention and control groups was 0.45 mm (95%CI = −0.05 to 0.94 mm), whereas in the five split-mouth studies [35, 36, 39, 43, 44], the mean difference in CAL gain between the intervention and control groups was 1.20 mm (95%CI = 0.72 to 1.69 mm) (Fig. 6)

### Meta regression

Random-effect meta‑regression analyses were used to explore the possible sources of heterogeneity among the studies. A separate univariate meta-regression model utilizing GTR as a predictor was significant for CAL (β = 0.296, 95%CI = −1.759 to −0.673, *P =* 0.001), indicating that the use of GTR has a significant influence on study outcomes. This variable explained all model heterogeneity, with no significant residual heterogeneity. No significant influences were observed for study design or the type of control (*P* > 0.05 for each). The results of the meta-regression analyses are shown in Table 4.

**Table 4:** Univariate meta-regression analyses of potential sources of heterogeneity

Heterogeneity factor	Exp (β)	Std. err	t-value	*P*-value	95 % CI		Adjusted *R* ^2^ (%)	*I* ^2^_res_ (%)
					UL	LL		
Use of GTR	0.296	0.244	−4.990	0.001	−1.759	−0.673	100.00	7.48
Study design	0.476	0.366	−2.030	0.070	−1.557	0.072	38.36	55.39
Type of control								
Allograft	2.461	0.570	1.580	0.149	−0.389	2.190	10.06	64.10
Synthetic	1.316	0.452	0.610	0.558	−0.747	1.296		

Note: Xenograft dropped because of collinearity. Adjusted R^2^ (%) = Proportion of between-study variance explained. I^2^res (%) = % of residual variation due to heterogeneity

### Assessment of risk of bias

**Fig. 7:**
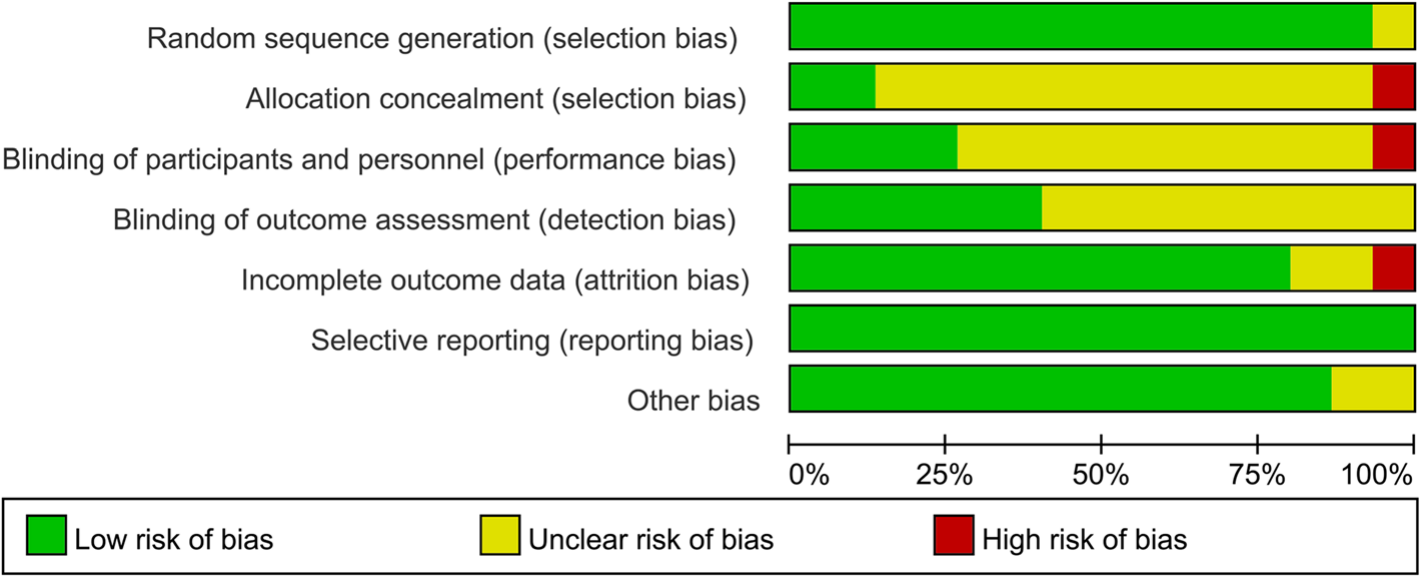
Risk-of-bias graph. The determinations made by the review authors regarding each risk-of-bias item are presented as percentages across all included studies

Of the included RCTs that evaluated the treatment of periodontal defects, three [11, 35, 40] were classified as having a low risk of bias, whereas ten [34, 36–39, 41–43, 45, 46] and two [44, 47] were determined to have a moderate and high risk of bias, respectively. The risk-of-bias graph presents a review of the authors’ judgments regarding each risk-of-bias item, and the values are presented as percentages across all included studies (Fig. 7).

## Discussion

The present systematic review aimed to assess the efficacy of PRP in the surgical treatment of periodontal intrabony defects based on randomized trials and reports that the use of PRP as an adjunct to a graft procedure yielded a significantly greater CAL gain and a PD reduction compared with the control treatment. Subgroup meta-analyses showed that the level of CAL gain was significantly higher in patients who were not treated with the GTR technique than in those who were. Moreover, a significant difference in observed outcomes was found for different study designs. We performed a meta-regression analysis to assess how the use of GTR, different study designs and the type of control affected heterogeneity. Only the use of GTR, which explained 100 % of the heterogeneity among the studies that assessed CAL gain, was identified as a source of heterogeneity.

In this study, we used a random-effects model for the meta-analysis, which assumed that the true effects were normally distributed. Overall, as determined primarily based on the results of the primary outcome variable (change in CAL), four of the RCTs [44–47] demonstrated that the addition of PRP to a specific GTR technique, i.e., β-TCP + GTR (e-PTFE) [44, 47], ABB + GTR (COL) [45], or NBM + GTR (COL) [46], failed to provide a statistically significant additive benefit to the management of periodontal intrabony defects. However, other RCTs [11, 34–43] reported that such adjunctive positive outcomes may result from the combination of PRP with other treatments, specifically HA [34, 11], BDX [35], ABB [36, 37], or DFDBA [39, 40]. A possible explanation for this finding may be that the control group (bone substitute + GTR) achieved a remarkable CAL gain and PD reduction and that the potential positive influence of PRP may be masked by the significantly high contribution of the regeneration materials on the clinical outcomes. Our results also confirmed the findings of other studies [12, 17]. In addition to serving as an indicator of positive outcome of periodontal regenerative procedures, PD reduction could also represent an important parameter in patient care because it directly reflects the ability to evaluate a treated area during maintenance appointments. According to the outcome variable (change in PD reduction), PRP therapy had a significant positive effect on periodontal intrabony defect treatment (the mean difference in PD reduction was 0.53 mm, 95%CI = 0.22 to 0.85 mm, *P =* 0.001).

Our analysis showed a significant difference between studies adopting a split-mouth design and those adopting a parallel design, and this finding indicates that different study designs are not equally effective in assessing the clinical efficacy of PRP. The attractiveness of the split-mouth design is the substantial reduction of inter-subject variability from the estimates of the treatment effect. However, the parallel-group design, in which all sites of one individual receive the same randomized treatment, is not only the simplest but also the most popular design used in clinical trials. A parallel design should be endorsed for the statistical comparison of outcome variables (i.e., CAL gain, PD reduction, and radiographic bone level) between the experimental and control groups. In contrast to the recommendations by *Lesaffre* et al. and the Cochrane Oral Health group, most systematic reviews did not evaluate split-mouth and parallel-arm trials separately in subgroup analyses [50]. *Smaïl-Faugeron* et al. reported a meta-epidemiological study that did not provide sufficient evidence for systematic differences in intervention effect estimates between split-mouth and parallel-arm RCTs for either continuous or binary outcome data [51].

The natural limitations involved in the preparation and application of PRP played an important role in evaluations of the efficacy of the adjunctive use of PRP in the management of periodontal intrabony defects. Some studies reported that differences in the level and the proportion of various growth factors may be detected using different commercially available systems and that this difference in the results may affect the outcomes [49, 52, 53]. Commercially available PRP systems can enrich platelets by two- to five-fold or even up to 10-fold, which is higher than the platelet concentration in whole blood. Additionally, researchers have reported that the effect of PRP is below the desired level at a low platelet concentration but that an inhibitory effect of PRP is observed at much higher PRP concentrations in whole blood [52, 53].

An important strength of our systematic review was the study selection because we used a range of databases and strict inclusion criteria for selecting the studies. There are also several limitations to this review. First, in this systematic review, we failed to undertake manual searches or identify unpublished research. It has been reported [54, 55] that the exclusive use of electronic data sources may not be a sufficient search strategy. This limitation may lead to a selection bias. Second, the majority of the RCTs incorporated an appropriate methodological approach, such as the definition of inclusion/exclusion criteria, the selection of suitable control groups, and appropriate methods of statistical interpretation. However, most of the RCTs had not performed sample size estimation before beginning their studies, which limits the evaluation of autologous PRP efficacy. Third, despite these findings, many of the RCTs selected in our analysis included small population sizes; therefore, additional large-scale clinical trials are required to clarify the long-term benefits of PRP. Additional research in this field is needed to consider specific factors, including sample size calculation and the allocation concealment and blinding methods used. Future studies planning to assess the adjunctive use of PRP in the treatment of periodontal intrabony defects should pay special attention to aesthetics, the rate of wound healing, and a subjective index as outcome variables because few of the selected RCTs reported these types of data.

## Conclusion

The adjunctive use of PRP together with conventional grafting procedures may be a beneficial treatment approach. However, when combined with the use of a regenerative technique, such as GTR, the beneficial effect of PRP on the treatment of intrabony defects is negligible.

## Abbreviations

ABM, anorganic bone mineral; BDX, bovine-derived xenograft; CAL, clinical attachment level; DFDBA, demineralized freeze-dried bone allograft; EGF, epithelial growth factor; FGF-β, fibroblast growth factor-β; GTR, guided tissue regeneration; HA, hydroxyapatite; HCP, human cultured periosteum; IGF-1, insulin-like growth factor-1; IGF-2, insulin-like growth factor-2; OFD, open flap debridement; P-15, peptide-15; PD, probing depth; PDGF, platelet-derived growth factor; PGFs, polypeptide growth factors; PRP, platelet-rich plasma; RCTs, randomized controlled clinical trials; TGF-1, transforming growth factor-1: TGF-2, transforming growth factor-2; TGF-β, transforming growth factor-β; VEGF, vascular endothelial growth factor
